# Determinants of early skeletal relapse after SSRO advancement in high-angle patients with mandibular retrognathia: A retrospective study

**DOI:** 10.1016/j.jpra.2026.03.023

**Published:** 2026-03-19

**Authors:** Hiroshi Nishioka, Makiko Yamauchi, Suguru Kondo, Yoshikazu Inoue, Takayuki Okumoto

**Affiliations:** Department of Plastic and Reconstructive Surgery, Fujita Health University School of Medicine, 1-98 Dengakugakubo, Kutsukake-cho, Toyoake, Aichi 470-1192, Japan

**Keywords:** Mandibular retrognathia, Sagittal split ramus osteotomy, Skeletal relapse, Mandibular plane angle, Genioplasty, Cephalometric analysis

## Abstract

**Background:**

Skeletal relapse after mandibular advancement via sagittal split ramus osteotomy (SSRO) remains a major concern in mandibular retrognathia. While advancement magnitude is frequently implicated, the independent contributions of Frankfort mandibular angle (FMA) and concomitant genioplasty remain debated.

**Methods:**

This retrospective study included 37 consecutive patients with mandibular retrognathia who underwent SSRO with or without genioplasty (January 2020–December 2024). Lateral cephalograms were obtained preoperatively and at 1 week and 6 months postoperatively. Using the Frankfort horizontal plane and a vertical reference plane through Sella, horizontal and vertical positional changes of B-point, Pogonion, and Menton were measured. Surgical movement was defined as the preoperative to 1-week change; relapse as the 1-week to 6-month change. Multivariate linear regression models included movement magnitude, genioplasty, and preoperative FMA. Exploratory models tested movement × FMA and movement × genioplasty interactions.

**Results:**

The cohort was predominantly female (36/37) with a mean age of 28.6 ± 6.4 years and a high mean FMA of 37.0 ± 2.8° Advancement magnitude was the only significant predictor of relapse, at B-point (horizontal and vertical) as well as at Pogonion and Menton (horizontal) (all *p* < 0.05). Neither FMA nor genioplasty showed independent effects. Interaction terms were not significant; however, visualization suggested a steeper relapse-advancement slope at higher FMA (40° vs 30°).

**Conclusions:**

In early follow-up, mandibular advancement magnitude was the primary determinant of skeletal relapse after SSRO for mandibular retrognathia, whereas FMA and genioplasty were not significant independent predictors. However, exploratory visualization suggested a possible advancement–FMA interaction, warranting larger studies to clarify potential effect modification by skeletal pattern.

## Introduction

Mandibular retrognathia is commonly treated using sagittal split ramus osteotomy (SSRO), yet postoperative skeletal relapse remains one of the most challenging issues in orthognathic surgery.^1^, ^2^, ^3^ Numerous studies have examined factors influencing stability, but reported outcomes vary widely. While several authors have identified the magnitude of surgical advancement as the strongest and most consistent predictor of relapse, the effects of other factors such as Frankfort mandibular angle (FMA) and concurrent genioplasty remain controversial.^2^^,^^4^

High-angle patients have been reported to show greater relapse and a higher risk of condylar changes after mandibular advancement.^5^^,^^6^ However, other studies, particularly those considering the amount of surgical advancement, have not consistently identified the FMA as a major determinant of postoperative stability.^4^^,^^7^ Similarly, although genioplasty has been suggested to influence postoperative muscle balance, recent analyses indicate little to no additional destabilizing effect.^7^^,^^8^ Previous studies have reported mixed findings regarding factors influencing relapse after SSRO, partly because most analyses used simple comparisons or correlations rather than multivariate regression models. As a result, the independent effects of surgical movement, FMA, and genioplasty have not been properly analyzed.

Our institution regularly treats mandibular retrognathia patients, including numerous high-FMA cases, and we have observed variability in postoperative stability that existing reports do not fully explain. To improve understanding, we conducted a retrospective study using standardized cephalometric measurements and a multivariate linear regression approach to clarify the independent determinants of early skeletal relapse after SSRO advancement, specifically within high-angle predominant population with mandibular retrognathia.

## Methods

### Ethics and study population

This study was conducted at Fujita Health University and approved by the institutional ethics committee, and written informed consent was obtained from all patients (HM25-302). We retrospectively reviewed patients with skeletal mandibular retrognathia who underwent SSRO either alone or in combination with genioplasty at our institution between January 2020 and December 2024. A total of 37 consecutive patients met the inclusion criteria. Demographic characteristics, preoperative cephalometric values, and operative details, including whether genioplasty was performed, were obtained from the medical record.

### Cephalometric acquisition and measurements

**Figure 1:**
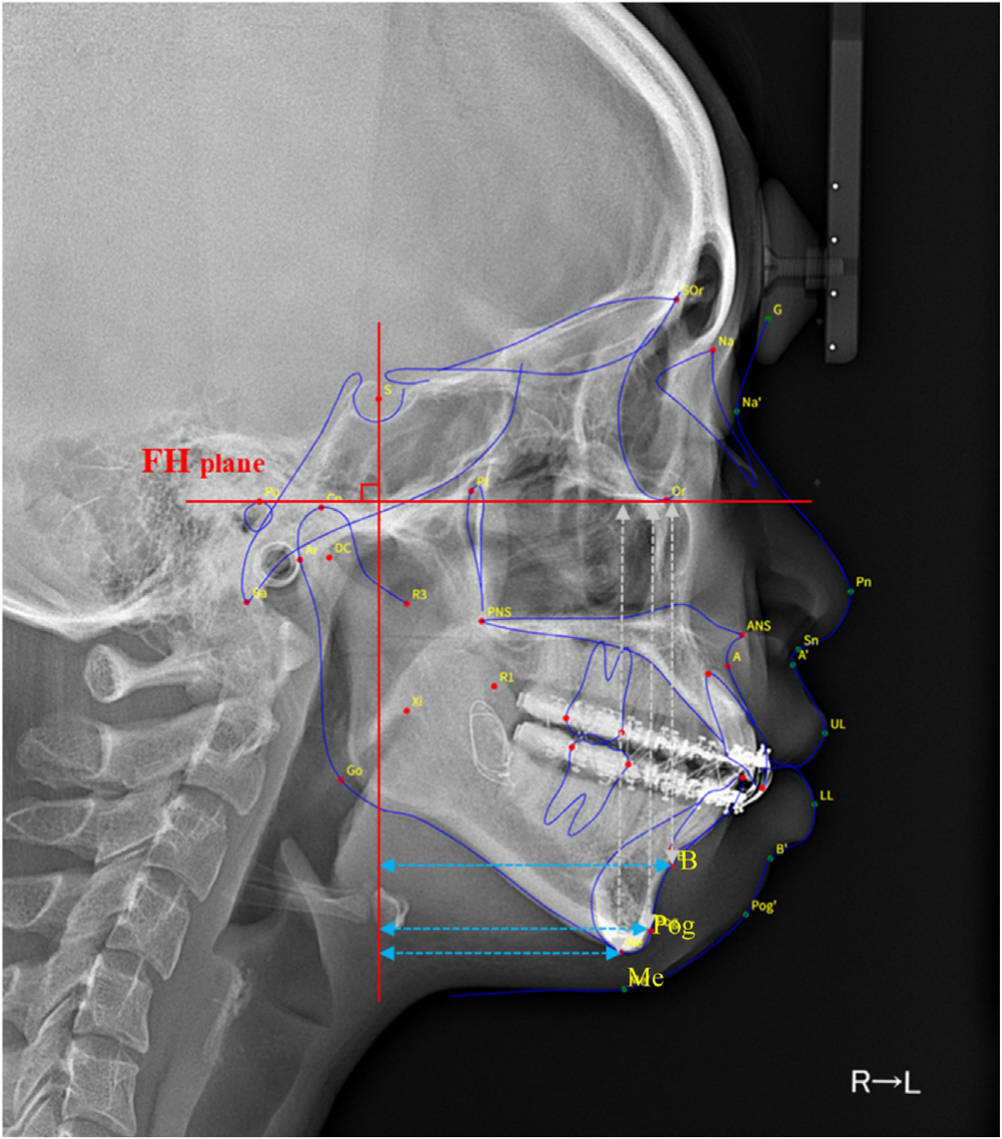
Cephalometric reference planes and landmarks used for analysis. The Frankfort horizontal (FH) plane and a perpendicular vertical plane through Sella were used as reference axes. Horizontal and vertical distances from B-point, Pogonion, and Menton to each plane were measured.

Standardized lateral cephalograms were taken at three time points: preoperatively, 1 week postoperatively, and 6 months postoperatively. Using the Frankfort horizontal (FH) plane and a vertical reference plane drawn perpendicular to the FH plane through Sella, horizontal and vertical distances from B-point, Pogonion, and Menton to each reference plane were digitally measured (Figure 1). This cephalometric coordinate system has been widely used in previous studies investigating skeletal relapse following mandibular advancement and allows standardized assessment of horizontal and vertical skeletal changes.^4^^,^^9^ Surgical advancement was defined as the positional change between the preoperative and 1 week postoperative cephalograms, whereas postoperative relapse was defined as the positional change between the 1 week and 6 month postoperative measurements.

### Primary statistical analysis

To identify independent predictors of skeletal relapse, multivariate linear regression analyses were performed with relapse as the dependent variable. The primary model included surgical movement, presence or absence of genioplasty, and preoperative FMA as explanatory variables. Separate models were constructed for the horizontal and vertical changes of B-point, Pogonion, and Menton. A *p*-value of <0.05 was considered statistically significant.

### Exploratory interaction analysis

In addition to the primary analysis, exploratory interaction analyses were conducted to assess whether the influence of surgical advancement on relapse varied according to skeletal pattern or concurrent genioplasty. For this purpose, interaction terms, movement × FMA and movement × genioplasty, were added to secondary regression models. All statistical analyses were conducted using R software.

## Results

Thirty-seven patients with mandibular retrognathia were included in this study, consisting of 36 females and 1 male, with a mean age of 28.6 ± 6.4 years. SSRO alone was performed in 21 patients, while 16 patients underwent SSRO combined with genioplasty. The mean FMA was 37.0 ± 2.8° (median 37°; IQR 32.8–41.3°), reflecting a high-angle–predominant cohort.

The surgical movements achieved are summarized as follows. The mean horizontal advancement at B-point was 3.9 ± 3.6 mm (median 4.4 mm; IQR 1.9–5.8 mm), and the mean vertical change was 2.5 ± 2.1 mm (median 2.0 mm; IQR 1.5–3.7 mm). At Pogonion, the mean horizontal advancement was 5.6 ± 4.8 mm (median 4.8 mm; IQR 1.9–8.6 mm), and the mean vertical change was 2.3 ± 1.6 mm (median 2.3 mm; IQR 1.1–3.3 mm). At Menton, the mean horizontal advancement was 5.6 ± 5.1 mm (median 4.9 mm; IQR 1.4–8.9 mm), and the mean vertical change was 3.6 ± 1.6 mm (median 3.4 mm; IQR 2.4–4.7 mm).

At 6 months postoperatively, skeletal relapse was observed at all landmarks. At B-point, the mean horizontal relapse was −1.6 ± 2.8 mm (median −1.2 mm; IQR −3.5 to 0.2 mm), and the mean vertical relapse was −0.8 ± 2.0 mm (median −0.6 mm; IQR −2.0 to 0.2 mm). At Pogonion, the mean horizontal relapse was −1.6 ± 2.8 mm (median −1.3 mm; IQR −4.0 to 0.3 mm), and the mean vertical relapse was −0.6 ± 1.6 mm (median −1.0 mm; IQR −1.5 to 0.2 mm). At Menton, the mean horizontal relapse was −1.5 ± 2.8 mm (median −1.6 mm; IQR −2.9 to 0.2 mm), and the mean vertical relapse was −0.9 ± 1.5 mm (median −1.0 mm; IQR −1.7 to 0.2 mm).

**Figure 2:**
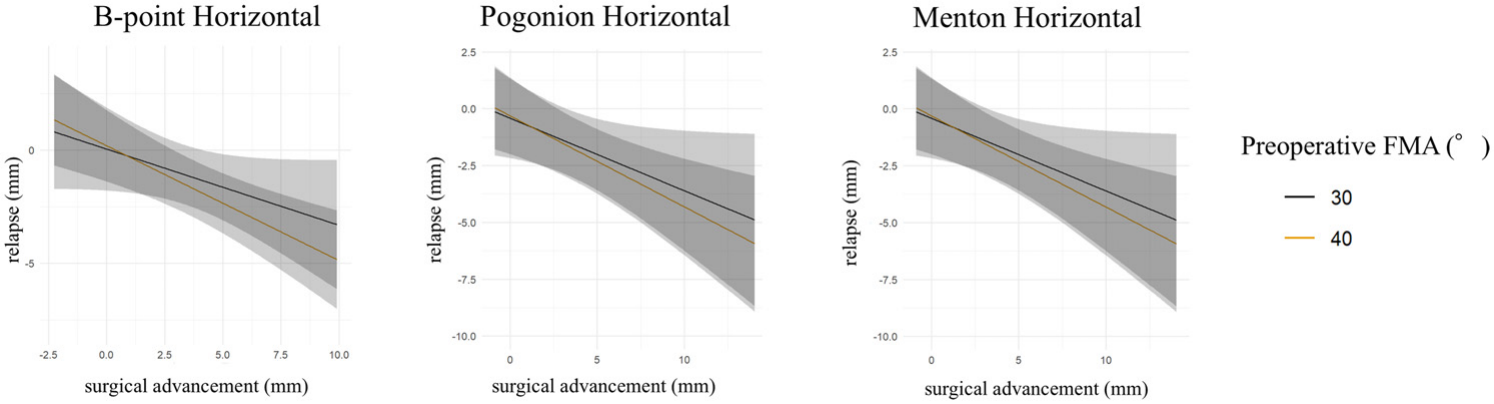
Predicted horizontal relapse according to surgical advancement magnitude and preoperative FMA (30° vs 40°), stratified by genioplasty. Shaded areas represent 95% confidence intervals. These lines represent predicted values based on the regression model, intended to visualize modeled interaction trends.

Multivariate linear regression analysis found that the magnitude of surgical advancement was the only significant predictor of postoperative skeletal relapse. Greater advancement was significantly associated with increased relapse at B-point in both horizontal and vertical directions, as well as at Pogonion and Menton in the horizontal direction (all *p* < 0.05) (Table 1, Table 2, Table 3). In contrast, neither preoperative FMA nor the presence of concomitant genioplasty showed a statistically significant independent effect on relapse at any landmark. Exploratory interaction analyses revealed no statistically significant interaction between surgical advancement and FMA or between surgical advancement and genioplasty. However, visualization at representative FMA values (30° vs 40°) showed a steeper predicted horizontal relapse–advancement slope at higher FMA (Figure 2). Although the interaction term was not statistically significant, this suggests a trend toward greater horizontal relapse with increasing advancement in high-angle patients.

**Table 1:** Multivariate linear regression analyses of skeletal relapse at B-point.

B-point vertical				
Predictor	B	SE	95%CI	*p*
Intercept	2.11	2.25	[−2.47 to 6.68]	0.355
Genioplasty	0.23	0.87	[−1.54 to 2.00]	0.797
Surgical movement	−0.46	0.13	[−0.72 to −0.21]	**<0.001**
Preoperative FMA	−0.05	0.06	[−0.18 to 0.07]	0.400

B-point vertical				
Predictor	B	SE	95%CI	*p*
Intercept	0.50	1.54	[−2.63 to 3.64]	0.746
Genioplasty	0.47	0.57	[−0.70 to 1.63]	0.418
Surgical movement	−0.58	0.13	[−0.84 to −0.32]	**<0.001**
Preoperative FMA	−0.002	0.04	[−0.09 to 0.08]	0.954

Bold values indicate statistical significance (*p* < 0.05).

**Table 2:** Multivariate linear regression analyses of skeletal relapse at Pogonion.

Pogonion horizontal				
Predictor	B	SE	95%CI	*p*
Intercept	0.97	2.40	[−3.92 to 5.85]	0.689
Genioplasty	2.09	1.14	[−0.24 to 4.42]	0.077
Surgical movement	−0.38	0.12	[−0.63 to −0.13]	**≤0.01**
Preoperative FMA	−0.04	0.07	[−0.17 to 0.10]	0.594

Pogonion vertical				
Predictor	B	SE	95%CI	*p*
Intercept	0.24	1.70	[−3.23 to 3.70]	0.890
Genioplasty	0.67	0.56	[−0.48 to 1.82]	0.243
Surgical movement	−0.30	0.17	[−0.66 to 0.05]	0.092
Preoperative FMA	−0.01	0.04	[−0.10 to 0.08]	0.803

Bold values indicate statistical significance (*p* < 0.05).

**Table 3:** Multivariate linear regression analyses of skeletal relapse at Menton.

Menton horizontal				
Predictor	B	SE	95%CI	*p*
Intercept	0.45	2.39	[−4.42 to 5.32]	0.851
Genioplasty	1.89	1.10	[−0.35 to 4.13]	0.096
Surgical movement	−0.34	0.11	[−0.57 to −0.11]	**≤0.01**
Preoperative FMA	−0.03	0.07	[−0.16 to 0.11]	0.709

Menton vertical				
Predictor	B	SE	95%CI	*p*
Intercept	−0.23	1.53	[−3.34 to 2.87]	0.879
Genioplasty	1.29	0.65	[−0.03 to 2.60]	0.055
Surgical movement	−0.23	0.19	[−0.62 to 0.15]	0.231
Preoperative FMA	−0.01	0.04	[−0.09 to 0.07]	0.796

Bold values indicate statistical significance (*p* < 0.05).

## Discussion

### Principal findings

In this retrospective cohort with 6-month follow-up, the magnitude of mandibular advancement was the primary predictor of early skeletal relapse after SSRO for mandibular retrognathia. Greater advancement was associated with greater relapse at B-point (horizontal and vertical) and at Pogonion and Menton (horizontal). In contrast, preoperative FMA and concomitant genioplasty were not significant predictors in the primary multivariable models. Exploratory interaction testing did not identify statistically significant movement × FMA or movement × genioplasty effects, although visualization suggested a steeper predicted horizontal relapse–advancement slope at higher FMA.

### Advancement magnitude and relapse

The association between greater advancement and increased relapse is consistent with prior studies identifying surgical movement magnitude as the most reproducible predictor of postoperative stability after mandibular advancement.^2^^,^^3^^,^^9^^,^^10^ Proposed explanations include increased tension in surrounding soft tissues and altered loading across the temporomandibular joint with larger advancements, which may contribute to postoperative remodeling and instability in susceptible patients.^11^^,^^12^ While some reports have proposed practical thresholds (often ∼6–7 mm) beyond which relapse becomes more frequent, such values should be interpreted cautiously because thresholds are sensitive to cohort composition, follow-up duration, and analytic approach.^2^^,^^9^ In this context, the present findings reinforce the clinical importance of carefully considering advancement magnitude during treatment planning and counseling.

### FMA and genioplasty

The role of FMA in relapse after advancement remains controversial. Several studies suggest that high-angle skeletal patterns are associated with greater relapse and/or condylar changes, whereas others report limited independent influence once the magnitude of advancement is considered.^4^, ^5^, ^6^^,^^8^^,^^12^ In the present study, FMA was not a significant independent predictor in the primary regression models. However, exploratory visualization at representative FMA values (30° vs 40°) suggested a steeper predicted horizontal relapse–advancement slope at higher FMA. Although the corresponding interaction term was not statistically significant—which may reflect limited statistical power in a relatively small cohort—this pattern is consistent with the possibility that the high-angle skeletal pattern may modify the response to larger advancements rather than exerting a strong main effect on relapse.

Concomitant genioplasty was also not a significant predictor of relapse, and exploratory analyses did not suggest meaningful effect modification by genioplasty. This aligns with prior work indicating that, when accounting for mandibular movement, concomitant genioplasty has little additional impact on skeletal stability after SSRO advancement.^4^^,^^7^^,^^13^ Taken together, the present findings and prior reports suggest that concomitant genioplasty does not materially influence early mandibular relapse in this setting.

### Clinical implications

Postoperative instability after mandibular advancement may be influenced by condylar remodeling or resorption, with both surgical and non-surgical risk factors reported, including advancement magnitude, skeletal pattern, and patient susceptibility.^11^^,^^14^^,^^15^ Fixation strategy is another important consideration, as increased rigidity does not necessarily ensure long-term stability and may predispose to unfavorable condylar seating.^16^, ^17^, ^18^ Accordingly, our institutional approach—Dal-Pont SSRO to maximize bony contact, intentionally loose or semi-rigid fixation, 3 weeks of intermaxillary fixation, and subsequent use of a functional surgical splint—aims to minimize biomechanical stress and facilitate physiologic adaptation. For high-angle patients, mandibular advancement at our institution is generally limited to 5 mm or less. This conservative strategy is supported by our present data, in which graphical analysis suggests that advancements exceeding approximately 5 mm are associated with a higher likelihood of clinically relevant relapse (≥2 mm). While previous reports have suggested that advancements up to 7 mm may be acceptable, such thresholds may be more applicable to non–high-angle cases. 3,9 Therefore, a more cautious advancement strategy may be warranted in high-angle mandibular retrognathia.

### Limitations and future directions

Several limitations should be considered. First, the modest sample size (*n* = 37) may have resulted in a Type II error, potentially limiting the detection of smaller effects from FMA or genioplasty. Second, the striking female predominance reflects East Asian clinical demographics but may affect the generalizability of our findings to male patients. Third, the generally conservative advancement magnitude and the use of 2D cephalometry might have masked the impact of high-angle patterns or 3D skeletal adaptations. Finally, the 6-month follow-up focuses exclusively on early relapse. Future prospective studies with larger cohorts, 3D imaging, and longer follow-up are necessary to fully clarify these complex interactions and long-term stability.

## Conclusion

After SSRO advancement for mandibular retrognathia, advancement magnitude was the primary predictor of skeletal relapse, whereas FMA and concomitant genioplasty were not significant predictors in adjusted models. However, exploratory visualization suggested a possible modification of the advancement–relapse relationship by skeletal pattern, warranting larger studies.

## Ethical approval

All procedures performed in studies involving human participants were in accordance with the ethical standards of the institutional and/or national research committee and with the 1964 Helsinki declaration and its later amendments or comparable ethical standards.

## Patient consent

All patients provided informed written consent for the use of their images.

## Declaration of competing interest

The authors have nothing to disclose.
